# Drought and heat stress tolerance screening in wheat using computed tomography

**DOI:** 10.1186/s13007-020-00565-w

**Published:** 2020-02-13

**Authors:** Jessica Schmidt, Joelle Claussen, Norbert Wörlein, Anja Eggert, Delphine Fleury, Trevor Garnett, Stefan Gerth

**Affiliations:** 1 School of Agriculture, Food and Wine, The University of Adelaide, Glen Osmond, SA Australia; 2 Fraunhofer Development Center X-Ray Technology, Fürth, Germany; 3 Innolea, 6 chemin de Panedautes, 31700 Mondonville, France

**Keywords:** X-ray, High-throughput, Phenotyping, Yield, Seed morphology, Genetic diversity

## Abstract

**Background:**

Improving abiotic stress tolerance in wheat requires large scale screening of yield components such as seed weight, seed number and single seed weight, all of which is very laborious, and a detailed analysis of seed morphology is time-consuming and visually often impossible. Computed tomography offers the opportunity for much faster and more accurate assessment of yield components.

**Results:**

An X-ray computed tomographic analysis was carried out on 203 very diverse wheat accessions which have been exposed to either drought or combined drought and heat stress. Results demonstrated that our computed tomography pipeline was capable of evaluating grain set with an accuracy of 95–99%. Most accessions exposed to combined drought and heat stress developed smaller, shrivelled seeds with an increased seed surface. As expected, seed weight and seed number per ear as well as single seed size were significantly reduced under combined drought and heat compared to drought alone. Seed weight along the ear was significantly reduced at the top and bottom of the wheat spike.

**Conclusions:**

We were able to establish a pipeline with a higher throughput with scanning times of 7 min per ear and accuracy than previous pipelines predicting a set of agronomical important seed traits and to visualize even more complex traits such as seed deformations. The pipeline presented here could be scaled up to use for high throughput, high resolution phenotyping of tens of thousands of heads, greatly accelerating breeding efforts to improve abiotic stress tolerance.

## Background

Wheat (*Triticum aestivum* L.) is one of the most important crops worldwide, accounting for 20% of the total calories and proteins in the human diet [1]. Its global production reaches 757.6 million tonnes per year with an annual consumption of 734 million tonnes [2]. Wheat yields are increasingly affected by global climate changes raising concerns regarding future food security. To increase wheat yields the understanding and genetic dissection of quantitative traits, especially those related to yield and stress tolerance, are required [3, 4].

Abiotic stresses such as heat, drought and frost negatively affect grain yield by reducing grain number, grain size and single grain weight. However, how and which yield component a certain stress affects varies with its duration, intensity and timing [5–7]. For instance, the occurrence of a stress before and during anthesis reduces the number of grains per ear due to an increased seed abortion, whereas grain weight is hardly affected. In contrast, an abiotic stress occurring after anthesis does not influence grain number but reduces grain size and single grain weight by impeding grain filling [5, 7, 8]. Further differences in the stress symptoms arise from temporal variation in flowering between and within ears of a single plant [9, 10]. In order to identify genomic regions responsible for grain yield and stress tolerance, the precise analysis of the grain yield components per ear and along the ear is crucial.

Currently, yield data is obtained by machine threshing in the field [11] or hand threshing for pot experiments. In wheat, about 10% of the seeds are lost during machine threshing, of which 4 to 6% is due to broken seeds compared with 1% seed breakage in samples threshed by hand [12, 13]. Hand threshing, on the other hand, is laborious, costly, time-consuming, and particularly difficult for wild accessions and hulled landraces [14]. The evaluation of yield-related traits such as seed weight, seed size and seed number require additional labour and restricts the number of samples which can be processed [15–17]. This is problematic for genetic experiments consisting in studying hundreds or thousands different genotypes of large populations, gene banks or mutant collections.

In order to improve the efficiency and accuracy of grain yield components measurements in large experiments, a number of automated approaches have been developed in the last few years. For instance, hyperspectral and RGB imaging have been used for the high-throughput measurement of yield-correlated traits such as plant height, ground cover, above-ground biomass and growth dynamics [18–22]. However, these methods do not allow researchers to directly assess grain yield components. More recently, computed tomography, a well-established technique in medicine, has been successfully adapted to plant phenotyping, showing a greater potential compared to hyperspectral and RGB imaging. In fact, along with measuring visible traits such as tiller number [23], computed tomography was employed to gain new insights into root growth plasticity within soil [24–26] and the internal and outer characteristics of seeds, such as size, porosity, infections and cracks [27–30]. Computed tomography has also been applied to predict yield components in rice and wheat [15, 31, 32] but with technical limitations so far.

Given the potential of computed tomography in plant phenotyping, this study aimed to develop an automatic, non-destructive and accurate method to measure a wide range of wheat grain set characteristics under different abiotic stresses. Previously used X-ray scanners were limited by the size of the computed tomography instrument that enable to fit ears of a maximum length of 10 cm [31, 32]. Larger ears had therefore to be cut in half leading to potential losses of seeds, useful data regarding ear architecture and seed position and causing longer preparation time. Further, scanning times were prolonged with up to 40–80 min per wheat ear. To keep scanning times moderate, lower resolutions had to be used hindering the analysis of morphological variations such as germ deformations and estimation of the weight of smaller seeds (< 2.0 mm) which are important to calculate stress tolerance traits. In contrast, the system we used in our study features a high-resolution flat-panel detector with a pixel pitch of only 49.5 µm and about 2304 pixel in the horizontal direction. Thus, scanning the ears close to the detector enables a large field of view while maintaining a voxel sampling of 31.25 µm.

We used a computed tomography scanner with integrated helix function that allowed us to analyse four intact ears with lengths up to 20 cm (including awns) at the same time with a two- to three-times higher resolution. As a result, we increased our throughput and were able to accurately assess total seed weight per ear, seed number per ear, seed weight along the ear and single seed characteristics such as single seed weight, seed size, seed shape, seed surface area and physical density of seeds. Additionally, we were able to reconstruct morphological deformations which are symptomatic of wheat grown under drought and frost stress. We tested the method on material with a broad genetic diversity and under two abiotic stress regimes.

## Results

### Computed tomography measurements

Our aim was to develop a non-invasive method to automate grain set measurements of wheat ears exposed to different abiotic stresses. The measured parameters included total seed weight per ear, seed number per ear (> 2.0 mm), seed weight along the ear and single seed characteristics such as single seed weight, seed size (volume), seed shape (spherical ratio) and seed surface area. Total seed weight refers to the sum of the weight of all seeds per ear. The single seed weight is the weight of one individual seed. Seed weight along the ear was calculated as the sum of the measured weight of seeds at the particular position for all ears. Single seed characteristics were measured for each seed individually and then averaged per ear.

Ears of plants subjected to either drought or combined drought and heat stress were initially measured with a total of 8441 projections and a scanning time of ~ 70 min for four ears. To increase the throughput, a lower number of projections (3082 projections) was tested which produced images of similar quality and were still accurate enough for data acquisition and analysis. Scanning time decreased to ~ 25 min, resulting in a scanning time of 7 min per ear. These settings were used for routine scans.

### 3D reconstruction and seed trait extraction

After the image acquisition of the raw 2D projections each stack of helical computed tomography scans is reconstructed using a Filtered-Back-Projection-Algorithm developed within the Development of X-ray Center (Fraunhofer Institute of Integrated Circuits, Germany). This reconstruction algorithm is implemented following the principle described in Buzug et al. [33]. This algorithm normalize the output 3D volume via the automatic detection of the unattenuated intensity on each projection. Thus, variations in the primary beam intensity does not change the calculated absorption within a voxel. Additionally, the algorithm convert the same range of absorption values within the raw 3D volume to an unsigned 16-bit grey value volume. Doing so, the grey values within the resulting 3D dataset represent the physical absorption within the ears for the specific x-ray beam spectrum used during the measurement. Without this normalization approach in the Filtered-Back-Projection, it is not possible to use the apparent grey values as a measure of the absorption. Within this publication all grey values mentioned are reconstructed as described above. If the physical absorption is mentioned, the derived float value of the reconstruction algorithm is meant.

**Fig. 1:**
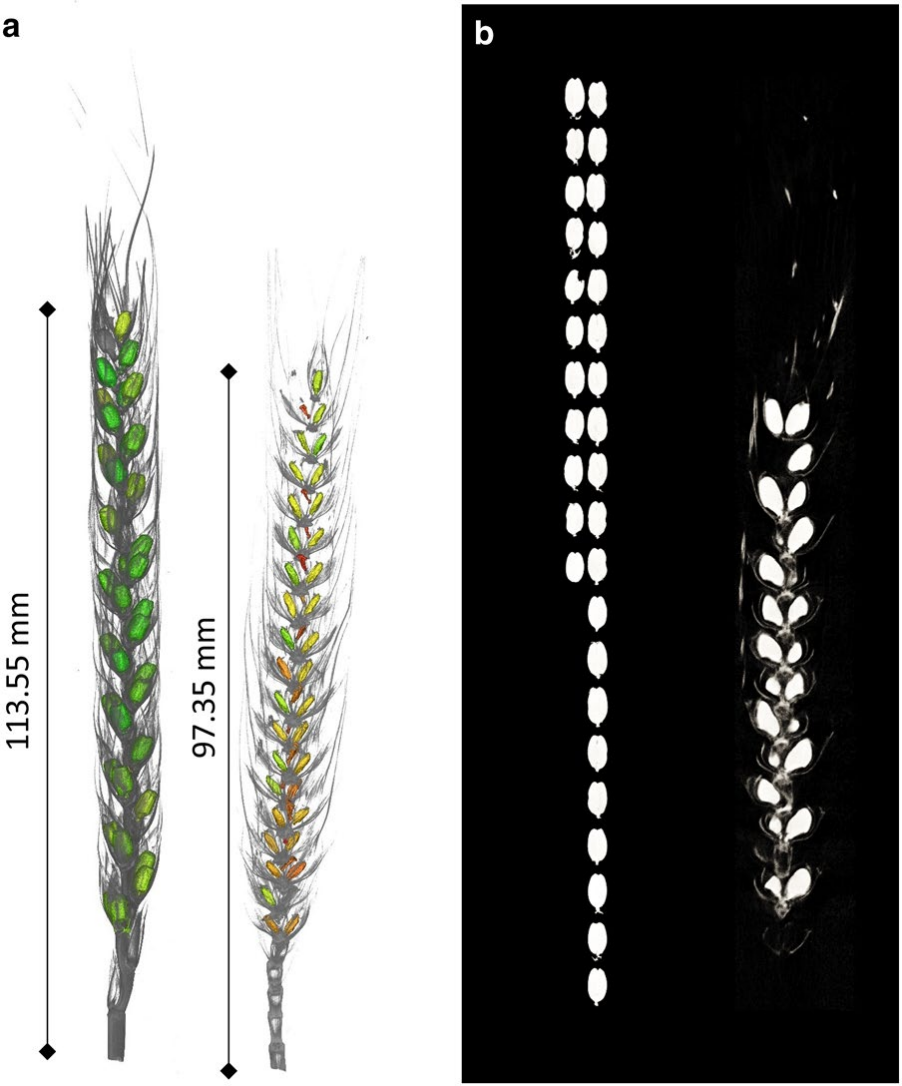
Reconstruction of wheat ears. **a** Virtual cross-section of the three-dimensional reconstruction of two wheat ears subjected to drought (left) and combined drought and heat (right) stress. **b** 2D representation of each seed at its maximum diameter of one ear

After the segmentation, the four ears within the volumes were manually divided into individual sub volumes. The EarS segmentation algorithm was used to segment the individual 3D volumes of an ear. An example of the visualization and segmentation is shown in Fig. 1. Additionally to the 3D visualization for two different ears (Fig. 1a), a 2D cross-section and the segmentation overview is shown (Fig. 1b). The algorithm is segmenting all seeds along the ear in 3D. Subsequently, each seed is stored in a small 3D volume for feature extraction. Additionally, a 2D cross section is created alongside the largest diameter within the seed (Fig. 1b).

Using a subset of about 10% ears, the correction factor between the virtual seed weight and the measured seed weight was determined. This correction factor was later on used for the calibration of the virtual total seed weight. For all the individual seed volumes a set of volumetric features is calculated and stored in a comma separated file.

### Evaluation of the image analysis algorithm

**Fig. 2:**
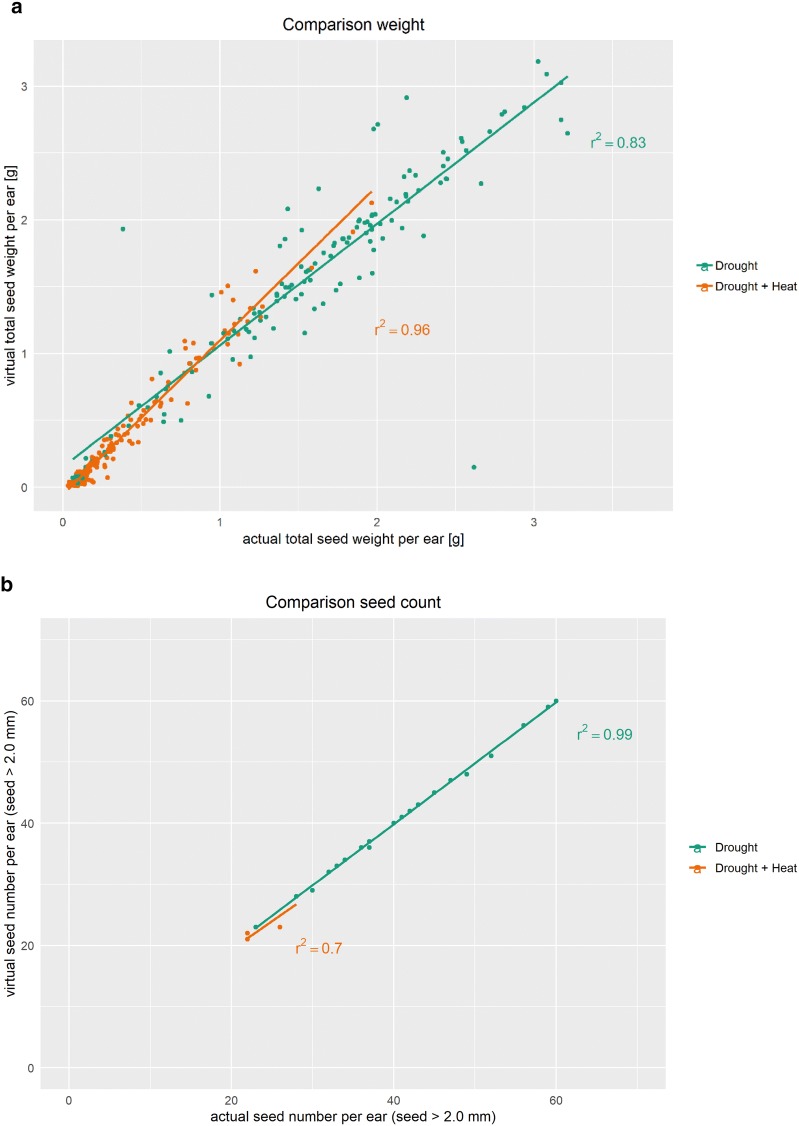
Predictions of seed weight and seed number per ear. Comparison of **a** virtual total seed weight per ear with actual total seed weight per ear and **b** comparison of virtual seed number per ear (seeds > 2.0 mm) with actual seed number of per ear. Under drought, 125 out of the 143 plants produced seeds above 2.0 mm, under combined drought and heat only 38 out of the 148 plants produced seeds of > 2.0 mm of size. r^2^, correlation coefficient

The performance of the algorithm was evaluated by comparing estimated (virtual) versus manually measured (actual) traits including total seed weight and number of seeds of > 2.0 mm size from the 291 wheat ears. The 2 mm diameter was selected to virtually represent the sieve during the threshing process. Thus, all seeds featuring a diameter smaller than 2 mm in every direction are omitted virtually. Scatter plots of virtual versus actual measurements are shown in Fig. 2a for the weight and 2b for the number of seeds > 2.0 mm. Total seed weight per ear was reduced (p ≤ 0.001) under combined drought and heat (DH) stress in comparison to the single drought stress (D). Seed weight under DH ranged from 0.03 to a maximum of 1.97 g per ear with an average of 0.40 g, whereas values ranged from 0.06 to 3.22 g with an average of 1.57 g per ear under D. Both plants with very small seed weight and with big seed weight were represented well by the algorithm, capturing a wide range of seed weights. Coefficients of determination (r^2^) were high between virtual and actual seed weight per ear with an r^2^ of 0.83 and 0.96 under D and DH, respectively.

Number of seeds > 2.0 mm of size were severely reduced (p ≤ 0.001) under DH in comparison to D. Under DH, only 25.7% of the plants contained seeds above 2.0 mm, whereas under D 87.4% of the plants produced larger seeds. In addition, plants under DH produced a maximum of 49 seeds per ear, while plants under D produced up to 73 seeds per ear. By contrast, the amount of small seeds (< 2.0 mm) per ear was increased under DH in comparison to D. The coefficient of determination between virtual and actual seed numbers were highest under D (r^2^ of 0.99). The coefficient of determination under DH were high (r^2^ of 0.70) but lower in comparison to D probably due to reduced number of plants containing big seeds.

Ears of the same accession but under different treatments showed mostly an average to good performance (i.e., seed weight ≥ 1.50 g, seed number ≥ 36) under D, but a bad performance under DH (i.e., seed number of zero). Ten out of the 46 accessions with ears exposed to D and DH were susceptible to both treatments with most of them being Australian varieties. Eight accessions performed relatively well under D and DH (i.e. seed weight ranging between 0.68 and 1.58 g with 17 to 45 seeds per ear) of which most were older varieties from Australia, Mexico, Asia and Canada. Even if some accessions performed well under both treatments, their performance under DH was never higher than the one under D which is not surprising since two stresses have a more severe impact on seed weight and seed number than a single stress alone.

### Virtual measurements of wheat grain set in response to drought and heat stress

**Fig. 3:**
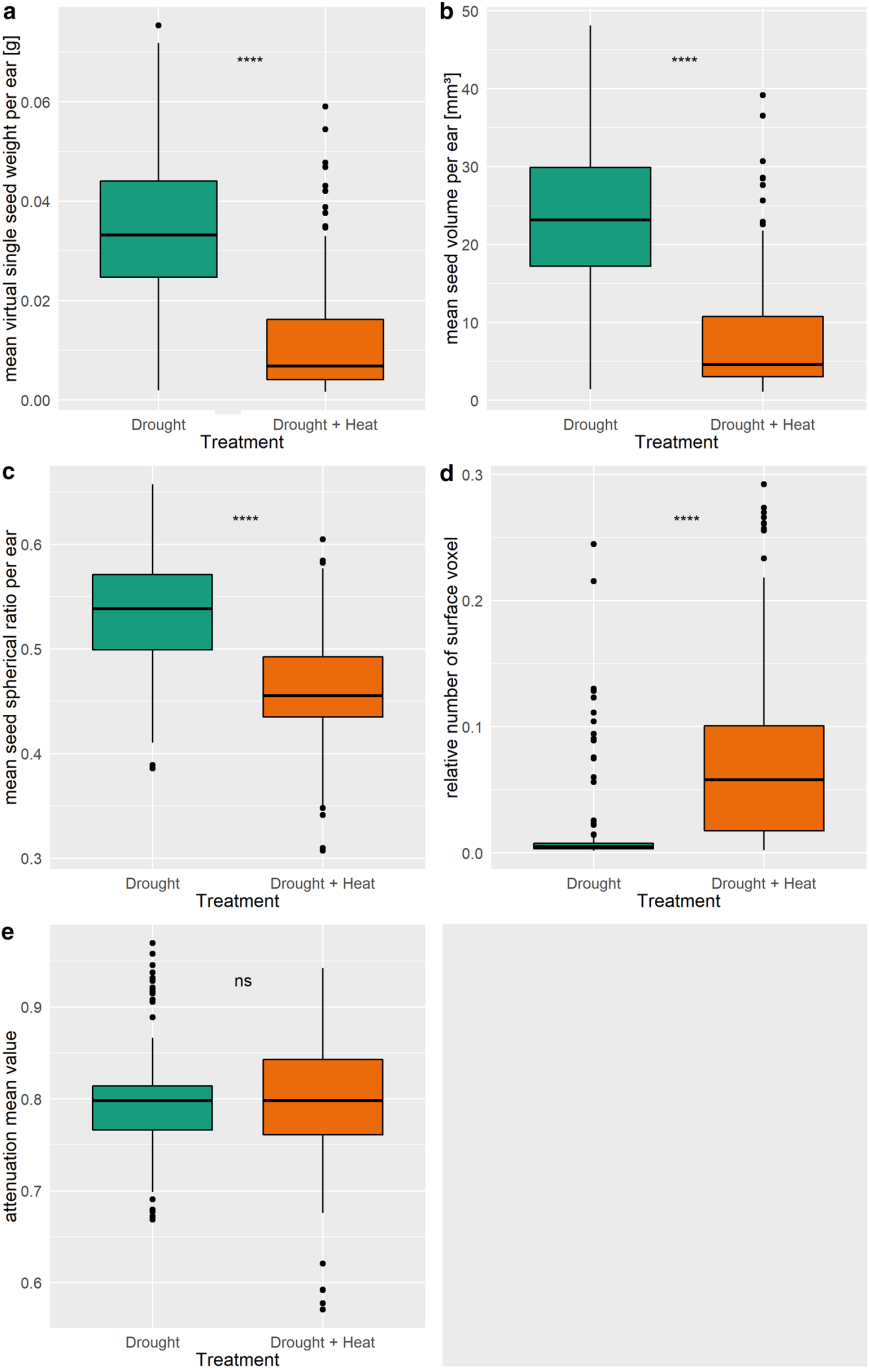
Virtually measured seed set characteristics under drought and combined drought and heat treatment. Mean attenuation mean value are representing the physical density of the seeds. Seed set characteristics were measured for each seed individually and subsequently averaged per ear. Drought: n = 143; drought and heat: n = 148. *, **, ***, ****Indicate p ≤ 0.05, 0.01, 0.001 and 0.0001, respectively. *Ns* not significant

The EarS algorithm was used to acquire seed set characteristics and to estimate the performance of diverse wheat accessions under two stress regimes. Significant differences between D and DH treatments were observed for four of the six virtually measured seed set characteristics (Fig. 3). Under DH, single seed weight as well as seed size (i.e. seed volume) were reduced in comparison to D. Seeds under D weight on average 0.03 g, whereas seed under DH were on average 0.02 g lighter, probably due to a reduced seed size. Seeds from plant under DH were also less round (i.e. reduced spherical ratio) but had an increased surface compared to seeds from plants subjected to D. Mean attenuation value is representing the physical density and were similar under both treatments with an average value of 0.80.

**Fig. 4:**
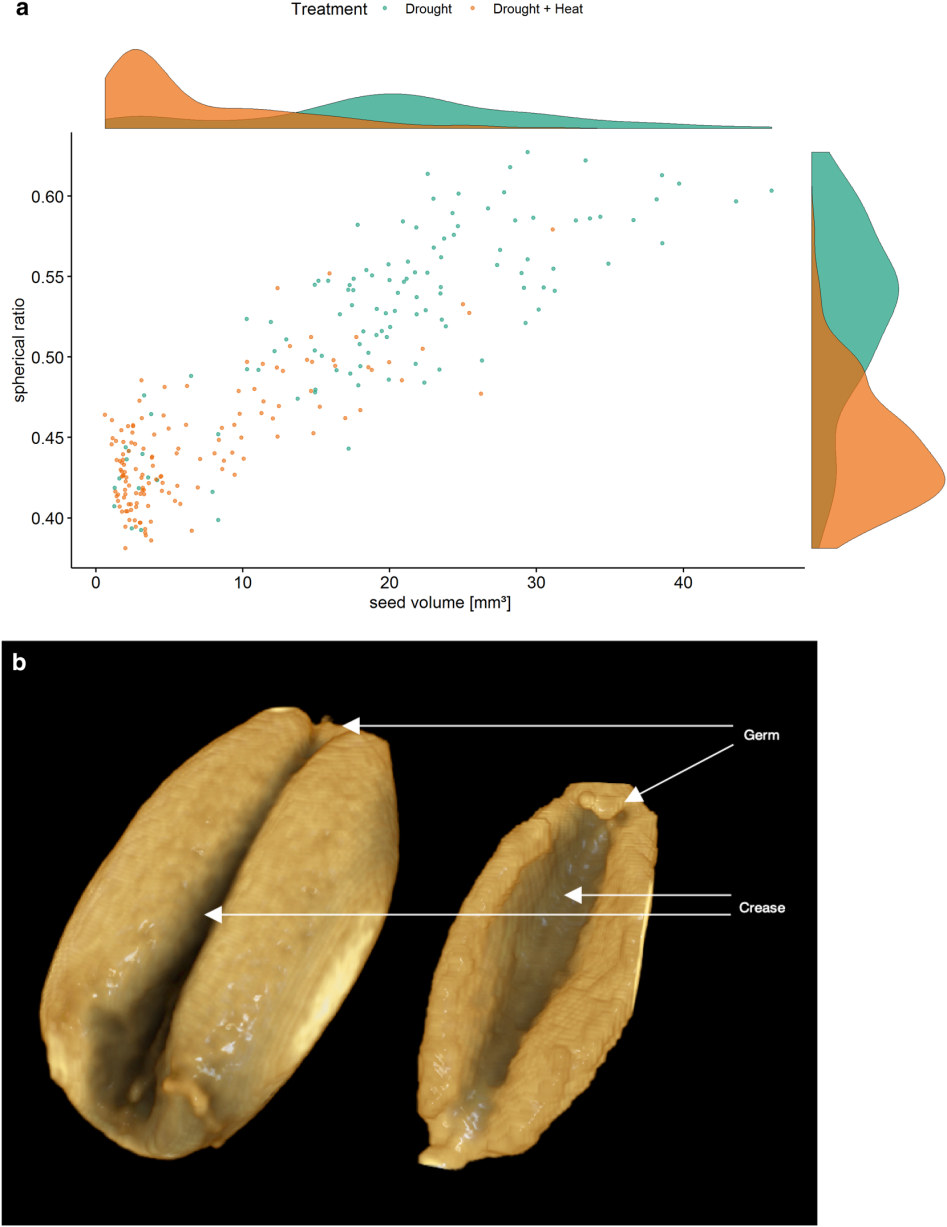
Seed morphology analysis. **a** Virtual spherical ratio versus virtual seed volume of single seeds under drought and drought and heat stress. **b** 3D reconstruction of a seed under drought stress with a length of 4.9 mm (left) and drought and heat stress with a length of 3.7 mm (right)

Decrease in spherical ratio of seeds under DH in comparison to D correlated with a decreased seed size, meaning that ears exposed to DH developed mostly seeds which were smaller and less round (Fig. 4). Ears of plants exposed to D contained mostly larger and rounder seeds with an average seed volume of 23.04 mm^3^ and a spherical ratio of 0.53 compared to an average seed volume of 7.98 mm^3^ and a spherical ratio of 0.46 under DH. Larger seeds had a fully developed crease and germ. The majority of the plants under DH showed severe seed deformations with large hollow cavities in the middle of the seeds (crease) and a not fully developed germ indicating an inhibition of grain filling or a late abortion. Those cavities lead to a larger surface area of seeds under DH compared to most seeds under D. The relative number of surface voxel is calculated as the ration of number of surface voxel divided by the volume of the seed. Which means that this value is close to zero for objects close to a sphere and close to one for a plane.

**Fig. 5:**
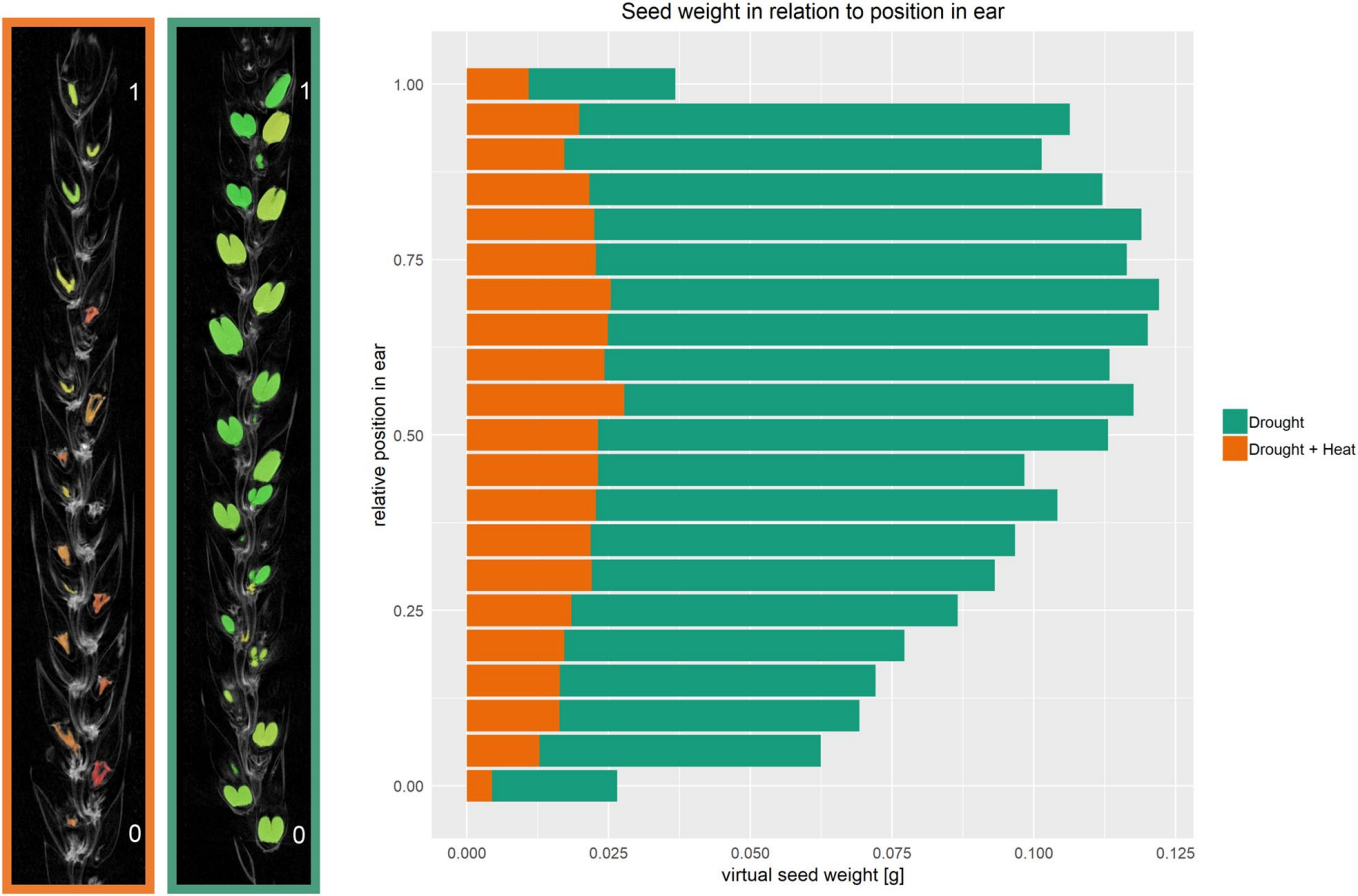
Virtual seed weight in relation to position in ear. 0 marks the first seed detected at the bottom of the ear including big (seed size > 2.0 mm) and small seeds (seed size < 2.0 mm) and 1 the last seed detected at the top of the ear. For illustration, a wheat ear subjected to drought (green background) and to drought and heat (orange background) are attached. Drought: n = 143; drought and heat: n = 148

To represent variations in the impact of both stress regimes on seed weight along the ear, the average virtual seed weight was plotted against the normalised ear position (Fig. 5). The central region of the ear contained more large seeds (seed size > 2.0 mm) than the top and bottom of the ear for both D and DH treatments. However, clear differences in seed weight between D and DH were observed. Under DH, seed weight was in general low along the whole ear with a slight decrease at the top and bottom of the ear (average 0.01 g) and the highest seed weight at position 0.55 (centre of ear, average 0.02 g). Average seed weight under DH was always below the seed weight under D along the whole ear (Fig. 5). Under D, average seed weight was severely decreased at the top and base of the ear (average 0.03 g) in comparison to rest of the ear. Interestingly, the highest seed weight was not located directly in the centre (position 0.50) but rather shifted towards the top of the ear (around 0.65–0.70, average 0.036 g).

## Discussion

Although the application of computed tomography to plants has been published before [23–32], our method enabled us to considerably increase speed and accuracy of grain measurement as well as to measure small genetic differences between wheats under stress conditions. Previously implemented computed tomography pipelines in wheat struggled with fusion problems between seeds due to the use of a low resolution. Further, due to long preparation and scanning times, only a small number of accessions and ears could be measured [30–32]. Our aim was therefore to develop a non-destructive, faster and more accurate computed tomography pipeline to predict yield related traits for a wide range of wheat accessions under different abiotic stress regimes.

Scanning time for each ear was around seven minutes in comparison to 40- and 80-min scanning time per ear in Hughes et al. [31] and Strange et al. [30], respectively, while using a two- to three-times higher resolution. The higher resolution allowed us to get a more detailed view of seeds and to reduce fusion issues achieving a higher accuracy of 0.83–0.96 for seed weight and 0.70–0.99 for seed number. In order to establish a computed tomography pipeline capable of predicting most accurately seed weight per ear and the derived seed traits, we calculated a virtual seed weight instead of seed volume only [31, 32]. Current pipelines tested in wheat work for fully developed, well filled seeds but would struggle to identify underdeveloped, poorly filled seeds. This is of importance due to the increased occurrence of underdeveloped seeds under abiotic stresses which feature a relative volume but are mainly hollow inside caused by the impediment in the development and grain filling of the seeds [34–36]. Poorly filled seeds affect negatively seed test weight and are diminished in their milling and baking qualities in comparison to well filled seeds. The differentiation between poorly and well filled seeds is therefore crucial in order to estimate the actual grain production and quality outcome [37].

Under D and DH, we detected small, underdeveloped seeds with a “shrivelled” appearance. This phenomenon has been previously observed visually in wheat by Cromey et al. [35] and Gaines et al. [37] under rainfed and frost conditions but has not been shown with any of the so far implemented computed tomography pipelines. Most shrivelled seeds occurred in plants exposed to DH indicating a synergistic effect of both abiotic stresses. Apart from the “shrivelled” appearance, we could also observe a stress induced deformation of the germ and an increased crease cavity explaining the increase in seed surface of smaller seeds (Fig. 4b). Future investigation of why and when these deformations occur might be possible using computed tomography scans gaining new insight into seed and ear morphology under different abiotic stresses. In contrast, the mean attenuation value was similar under both treatments, indicating that the starch density was only slightly affected by the heat component of the DH treatment. Smaller seeds seem therefore to accumulate starch in the same density but in less amount than bigger seeds causing potentially the described cavities.

As in previous studies, we could demonstrate the synergistic effect of D and H on seed weight, single seed weight and number of seeds > 2.0 mm [38, 39]. All traits were similarly affected with a decrease between 64 and 83%, the highest decrease being in seed number. Seed number explains up to 84% of the variation in yield and is therefore a crucial component for improving yield under abiotic stress [40–42]. Most studies report a decrease in seed number if the stress occurs during meiosis,however, recent papers showed also an effect on seed number under post-anthesis stress [38, 39, 43]. Average seed weight was decreased at the top and bottom of the ear under both treatments with a more severe effect of DH. The decrease of seed weight at the extremes of the ears is likely due to the spatial differences in the development of the ear with the middle flowering usually earlier than top and bottom parts [9, 10].

Using a genetically diverse material enabled us to capture a broad variation in seed weight and components for implementing our pipeline to identify germplasm with an increased drought and heat tolerance. In our study, eight accessions, predominantly from countries characterized by dry and hot summers (i.e., Australia, Iraq, Tunisia, India and Mexico) showed relatively good performances under both treatments in regard to seed weight. Those accessions produced on average more seeds and seeds of increased weight, size and rounder shape. Screening genetic diversity for tolerant germplasms is essential for mining novel candidate genes underlying traits of agricultural importance such as abiotic stress tolerance. Novel genes and alleles can then be implemented in breeding programmes to breed high-yielding crops adapted to future climates [44].

### Future perspectives

The combination of both molecular and high-throughput phenotyping technologies is important for the improvement of crops under abiotic stresses [45]. The computed tomography pipeline we developed enables the analysis of thousands of ears in a relative short time, numbers that would allow to study precisely the genetic of grain set development in large genetic populations and mutant collections. The approach could also be used to screen large and diverse collections preserved in gene banks allowing to explore distant gene pools and possibly preselect material with high yield potential for breeding. The method could also be adapted to other crops such as barley. Single time point measurements could also be replaced by dynamic data such as kinetics of grain growth and potentially embryo development as well as developmental alterations over time induced by abiotic stresses.

## Conclusions

We developed a robust, fast and accurate computed tomography pipeline for wheat which can measure seed traits under various stress conditions and for a broader range of wheat accessions. We demonstrated that our method can detect small genetic differences between wheats, ears and even single seeds, which is essential to improve grain yield and produce resilient varieties. More importantly our method is amenable to automation enabling us to phenotype, with high resolution, one hundred thousand wheat heads in 6 weeks only. This throughput is compatible with large scale genetic studies and breeding programmes where hundred thousand yield plots have to be assessed every year in the field. Additional morphological traits such as seed shrivelling and germ deformation have been measured for the first time under combined drought and heat stress. Our computed tomography pipeline is therefore a quick method enabling the measurements of common seed traits in combination with a detailed analysis of seed and ear morphology.

## Methods

### Plant material

A panel of 315 diverse bread wheat accessions was sown in 2017 (from May to November) on the Waite Campus of the University of Adelaide (Urrbrae, South Australia). Plants were grown in a polyurethane tunnel in pots filled with a substrate mix (clay-loam, sand and coco peat, 1:1:1) supplemented with a basal, slow-release fertilizer. Pots were watered from underneath and kept under well-watered conditions until anthesis. Three days after anthesis of the primary tiller, plants were exposed individually to either drought or combined drought and heat, mimicking growing conditions common in the southern Australian climate. Drought was imposed by stopping irrigation for 6 days. The heat treatment was applied on the 4th day of the drought treatment by moving the plants in a heat chamber to expose plants to 35/25 °C day/night for three days. Treatments were arranged in a randomized split-plot design with three replications. A subset of 291 ears of the primary tiller which varied regarding yield and yield-related traits (143 drought-treated and 148 combined drought and heat-treated) were selected from 203 of the 315 accessions with 46 accessions represented in both treatments. A list of accessions is given in Additional file 1.

### Computed tomography measurements

X-ray computed tomography measurements were performed at the Development Center X-Ray Technology (EZRT) of the Fraunhofer Institute of Integrated Circuits (Fürth, Germany) using the “CTportable160.90”. The scanner consisted of a cone beam X-ray source with voltage ranging from 20 to 90 kV, current up to 89 µA and a detector size of 2304 × 1278 pixels (49.5 μm pixel size). The system consists of a Thermo Scientific PXS5-928 microfocus monoblock X-ray source, featuring a transmission target with only 6 mm spot to window spacing. Additionally, the focal spot size of the source is dependent on the actual power (4 µm @ 2 watts). The sample stage can be positioned between X-ray source and detector with a minimum focus object distance (FOD) of 10 mm and a maximum FOD of 285 mm. The focus detector distance (FDD) is fixed to 290 mm. Thus, the maximum nominal magnification is 29 for objects with diameters below 8 mm. However, limiting the maximum magnification to about 18 will result in an acceptable edge blurring of 1.4 pixel due to the focal spot size of 4 µm. The resulting maximum resolution is 2.8 μm. The detector is a 14 bit CMOS sensor (Teledyne DALSA Shad-o-Box 3K HS) with a direct-contact Gd_2_O_2_S scintillator (Kodak Min-R 2190) scintillation foil.

Within this experiment, the system was operated at 90 kV and 80 µA and a nominal spatial voxel sampling of 31.25 µm to cover the biggest field of view during the scan. To extend the vertical field of view a helical scanning geometry was used to scan four ears simultaneously (i.e. vertical movement during several 360° rotations). Thus, ears were scanned in their full length and at the same time Feldkamp artefacts are avoided. A total of 3082 projections within 4.24 times 360° rotations in a helical scan were taken with an exposure time of 500 ms in a FlyBy acquisition allowing the system to rotate the ear constantly during the image exposure. Each scan lasted about 27 min producing images with a spatial voxel sampling of 31.25 μm pixel^−1^. The software Volex 6 (Fraunhofer Institute of Integrated Circuits, Germany) was used for controlling the system.

### 3D reconstruction and seed trait extraction

Images were 3D reconstructed with a filtered back projection (Fraunhofer Institute for Integrated Circuits, Germany, REL-2.1.1) and cropped using the software VolumePlayerPlus (Fraunhofer Institute for Integrated Circuits, Germany, 8.1.9). The algorithm EarS (Fraunhofer Institute for Integrated Circuits, Germany, Revision number 227116) determines a wide range of wheat ear and grain components. To evaluate the performance of the image analysis algorithm, ears were carefully threshed by hand and total seed weight and seed number (seeds > 2.0 mm of size) were measured. The obtained parameters were correlated with the data from the X-ray scans. The EarS algorithm first separates each individual grain from the ear itself. Each grain is automatically oriented using a principal axis transformation in 3D. Thus, 2D cross sections alongside the largest slice can be used for fast visual inspection of the performance of the algorithm. Additionally, for each individual grain different features are analysed in 3D and stored in a csv file. With the current revision number 227116 of EarS, for each grain, following digital traits are calculated within the ear:

- Amount of voxel in the grain.
- Corresponding volume of the grain in mm^3^.
- Virtual weight in g.
- Centre of mass of the grain in the 3D volume in x, y, z coordinates.
- Mean attenuation value of the grain.
- Aspect ratio of the grain.
- Surface of the grain.
- Ratio between volume and surface of the grain.

The amount of voxel is gathered by automatically separating the grain from the surrounding biological material. Having the voxel sampling size calibrated within the system, the volume of the whole grain is derived multiplying the physical voxel size with the amount of voxel within a seed. Using the volume and the mean physical absorption, a virtual weight can be calculated and therefore the position of the centre of the mass of each grain [46]. The value of the mean physical absorption is directly generated from the normalization of the FireFly Filtered-Back-Projection. The centre of mass is used to calculate the distances between individual grains alongside the ear. The surface is approximated by counting the number of voxels connected to the binary background of the volume. Out of this, it is possible to calculate the surface to volume ratio for each grain. Another morphological trait is the aspect ratio calculated by comparing the radius of the minimum covering sphere with the radius of a sphere featuring the same volume as the grain. Values between 0 and 1 are derived, where 0 would be an infinite long and thin rod, and 1, a perfect sphere. Whereas most of the calculations for the aspect ratio are in 2D [47], the EarS algorithm expands the aspect ratio calculation to 3D.

## Supplementary information

**Additional file 1.** List of* Triticum aestivum *accessions.

## Data Availability

Not applicable.

## Abbreviations

2D: Two-dimensional
3D: Three-dimensional
D: Drought
DH: Combined drought and heat stress
r^2^: Correlation coefficient
RGB: Red–green–blue
